# Design and Fabrication of a Fully-Integrated, Miniaturised Fluidic System for the Analysis of Enzyme Kinetics

**DOI:** 10.3390/mi14030537

**Published:** 2023-02-25

**Authors:** Andreas Tsiamis, Anthony Buchoux, Stephen T. Mahon, Anthony J. Walton, Stewart Smith, David J. Clarke, Adam A. Stokes

**Affiliations:** 1 School of Engineering, Institute for Integrated Micro and Nano Systems, The University of Edinburgh, The King’s Buildings, Edinburgh EH9 3FF, UK; 2 School of Engineering, Institute for Multiscale Thermofluids, The University of Edinburgh, The King’s Buildings, Edinburgh EH9 3LJ, UK; 3 School of Engineering, Institute for Bio-Engineering, The University of Edinburgh, The King’s Buildings, Edinburgh EH9 3FF, UK; 4 EaStCHEM School of Chemistry, The University of Edinburgh, Edinburgh EH9 3FJ, UK

**Keywords:** sensors, fluidics, integration, lab-on-a-chip, integrated devices, miniaturised total analysis system, optofluidics

## Abstract

The lab-on-a-chip concept, enabled by microfluidic technology, promises the integration of multiple discrete laboratory techniques into a miniaturised system. Research into microfluidics has generally focused on the development of individual elements of the total system (often with relatively limited functionality), without full consideration for integration into a complete fully optimised and miniaturised system. Typically, the operation of many of the reported lab-on-a-chip devices is dependent on the support of a laboratory framework. In this paper, a demonstrator platform for routine laboratory analysis is designed and built, which fully integrates a number of technologies into a single device with multiple domains such as fluidics, electronics, pneumatics, hydraulics, and photonics. This facilitates the delivery of breakthroughs in research, by incorporating all physical requirements into a single device. To highlight this proposed approach, this demonstrator microsystem acts as a fully integrated biochemical assay reaction system. The resulting design determines enzyme kinetics in an automated process and combines reservoirs, three-dimensional fluidic channels, optical sensing, and electronics in a low-cost, low-power and portable package.

## 1. Introduction

Lab-on-a-chip technologies have focused on miniaturising physical and chemical processes to enhance the understanding of biochemical systems. This can be achieved by integrating multiple discrete laboratory processes or techniques into a system that fits on a chip. The technology is particularly beneficial in the life sciences due to advantages over conventional laboratory testing, such as low cost due to reduced reagent volumes, reduced detection times, processing capabilities hundreds of times faster than current technologies, and high throughput with the parallelisation of processes [1,2]. However, the capabilities and commercial impact of such devices are often limited due to the additional bulky peripheral equipment, such as syringe pumps, microscopes, and power supplies, required for operation [3]. These integral components are often not integrated and omitted as requirements for scaled systems, limiting lab-on-a-chip technologies to operations within the laboratory.

Furthermore, routine laboratory analysis takes place in multiple domains (fluidic, electronic, pneumatic, hydraulic, photonic, etc.) and there has been limited integration of R&D outputs into total analysis systems. Systems built in the R&D arena which utilise high levels of integration could enable the delivery of breakthroughs in research, although incorporating multiple domains and all physical requirements into a single device remains a challenge. Manz et al. introduced the term miniaturised (micro) total analysis system, or µTAS, as a system that comprises a channel network wherein the channels have micrometre-scale dimensions, can be fabricated into or from a solid substrate, integrates multiple discrete laboratory functions on a single chip, and where the fluid flow in a microstructure is a required element of the analytical or preparative function of the device [4]. The authors outlined the scaling potential and benefits of miniaturising such systems. The term µTAS is less associated with devices nowadays and instead represents miniaturised systems for chemistry and life sciences through a popular conference of the same name. It should also be noted that total analysis systems are now in high demand for emerging technologies such as organ-on-a-chip [5,6,7]; a concept which has recently gained significant popularity among researchers.

This paper reports such a miniaturised, fluidic total analysis system used as a demonstrator platform. It details the design, fabrication, and operation of a single unit, automated device integrating several domains for measuring enzyme kinetics. The system integrates reservoirs, three-dimensional (3D) fluidic channels, sensing, and electronics in a low-cost, low-power, and portable package. The fluidics were fabricated using rapid prototyping techniques and following a modular development approach of key components that can be readily modified or adapted for use with other fluidic systems.

## 2. Background

Research on microfluidics has primarily focused on the development of components such as microvalves [8,9,10,11], mixers [12,13,14], and micropumps [15,16,17,18]. However, there are also notable fluidic devices. Sauer-Budge et al. demonstrated a complex fluidic network cartridge to identify bacterial pathogens in clinical samples [19]. The device could prepare different samples (urine, blood, and stool) for a polymerase chain reaction. However, this passive cartridge required a large (although single unit) tool to perform the analysis. Schumacher et al. demonstrated a complex integrated lab-on-a-chip with multiple capabilities and a user interface [20]. Components such as pumps, reservoirs, analysis chambers, and electrochemical sensors were integrated into a single device with fluidic and electrical interfaces. The fluidic components were fabricated with cycloolefin copolymer via mould injection, and the electronics included silicon-based sensors on a standard printed circuit board (PCB). Brassard et al. developed a centrifugal microfluidic platform with active pneumatic pumping for the integration and automation of sample preparation processes [21]. This was demonstrated by extracting nucleic acids from whole blood.

Microfluidics have taken advantage of a plethora of pre-existing fabrication technologies. The development of soft lithography by Duffy et al. [22] has helped to expand the field. This method is inserted into an already established framework of cleanroom microfabrication, using standard photolithographic techniques and wafer processing technologies. Scaled and mass production reduce typically significant upfront costs. Many devices fabricated in cleanroom facilities have lacked the integration of all the key equipment necessary for operation, although more recently Wang et al. used soft lithography to fabricate a lab-on-chip device for rapid nitrate determination, which was based on double microstructured assisted reactors [23]. 

By successively removing layers from a material, micromilling offers an alternative solution for fabricating fluidic networks. Metals, ceramics, and polymers are typical materials with chemical and temperature tolerances required for microfluidic devices. For example, Chen et al. used a high-speed spindle to fabricate fluidic devices in polycarbonate with an average wall roughness of 0.15 µm ± 0.08 µm [24]. Yen et al. demonstrated that micromachining can offer a cost-effective platform for the rapid prototyping of microdevices [25]. More recently, Lashkaripour et al. optimised the fabrication of microfluidic devices on polycarbonate using a low-cost desktop micromilling tool [26]. Patterns of reduced surface roughness with feature sizes as small as 75 µm were demonstrated.

Paper microfluidics is a low-cost technology, where channels are patterned on paper using a number of methods [27,28]. One such method is wax printing where the wax is melted and infuses through the paper to create hydrophobic barriers. Using this approach, fluidic devices can be designed and fabricated in minutes [29]. Low-cost and biodegradable materials are key factors for producing single-use devices, such as for blood sample analysis. Laser cutting is a further option [30,31]. For example, a capillary network with consistent channel depths was engraved on 2 mm thick acrylic, in order to study bubble lodgement in industrial and biological processes [32]. This complex capillary system mimicked physiological vascular networks with rectangular channels between 0.26 and 0.52 mm.

Briefly, 3D printing enables the rapid prototyping of complex fluidic devices [33,34]. For example, Kitson et al. fabricated chemical reactors with 800 µm channels, which were printed in polypropylene by fused deposition modelling (FDM) [35]. Anderson et al. [36] used stereolithography printing to fabricate a microfluidic device that also incorporated threaded ports for connecting to syringe pumps and cavities for cell culture inserts. The device included an array of eight channels, 3 mm wide and 1.5 mm deep. Similarly, Shallan et al. [37] produced a series of low-cost, complex 3D microfluidic devices, including a 3D mixer, a gradient generator, a droplet generator and a tool to analyse nitrates in tap water (the smallest fluidic channels were 500 × 500 µm).

Furthermore, 3D printing can also be combined with lost core methods. A negative mould of a fluidic network is printed, encased in a material such as epoxy or polydimethylsiloxane (PDMS) and subsequently removed, leaving a void. Therriault et al. developed such a method to create complex 3D structures in epoxy [38]. A matrix of channels 10–250 µm was initially printed using fugitive organic ink. This was then encased in epoxy and heated to 60 °C, melting the ink and creating the fluidic network. The network was filled with a photocurable resin, which was selectively cured using a set of photomasks, resulting in a complex chaotic mixer. Although the method allowed for channels as small as 10 µm, the design capabilities of the fabrication method are limited. A simpler lost core-based fabrication technique has been previously described by the authors of this paper [39]. Briefly, acrylonitrile butadiene styrene (ABS) is used as the material to FDM print the sacrificial mould of a complex 3D fluidic network, which is then encased in PDMS and finally immersed in an acetone bath. When placed in contact with acetone, ABS will dissolve. By taking advantage of the swelling properties of PDMS in solvents [40], any ABS embedded in PDMS is liquefied before being washed away, thus leaving a void corresponding to the designed fluidic network. Using a similar fabrication technique, Saggiomo et al. [41] demonstrated that electronics could be incorporated into a fluidic device before being encased in PDMS. The fabrication of the fluidics presented in this paper follows the lost core methodology developed in [39].

## 3. Materials and Methods

### 3.1. Enzyme Kinetics Analysis

To demonstrate the operation and capabilities of the proposed demonstrator fluidic device, a robust and inexpensive assay has been selected [42]. The selected enzyme, although well-characterised, retains clinical relevance, and the measurement of activity is typically included in routine blood work. In the presence of alkaline phosphatases (phosphatase, alkaline from bovine intestinal mucosa, Sigma-Aldrich) the reaction results in the transformation (by hydrolysis) of the colourless synthetic substrate, *p*-nitrophenyl phosphate (PNPP Substrate, Thermo Fisher Scientific) into a yellow-coloured product, *p*-nitrophenol. This provides the opportunity to integrate optical colour detection into the system.

The reaction mechanism is described by:

(1) E+S⇌ES→E+P

where an enzyme (E) and a substrate (S) are mixed to facilitate an enzyme–substrate complex (ES) that needs to be formed before the reaction can take place to form the product (P) and released by the free enzyme (E). Enzyme kinetics analysis is performed using the Michaelis–Menten model, which relates reaction rate or velocity (*v*) to the concentration of substrate ([S]) through:

(2) $$v=\frac{V_{max}\;\left[S\right]}{K_{m}+\left[S\right]}$$

where *V_max_* is the maximum reaction velocity at maximum (saturating) [S] and *K_m_* is the Michaelis–Menten constant, equal to [S] at which the reaction rate is half of *V_max_*.

For the described assay, the initial reaction rate is seen as a colour change in the mixture. Spectrophotometry in conjunction with Beer–Lambert’s law allows the determination of the reaction rates through the measurement of initial rates of change in light absorbance for a number of known sample concentrations (enzyme and substrate). This in turn enables *K_m_* to be determined.

This type of experiment is typically operated in many laboratories and requires the availability of external commercial equipment. The concentration of the substrate is manually varied using pipettes into multi-well plates while keeping the enzyme concentration constant. Thus, the accuracy of the results often depends on the skill of the operator. The procedure is ideal for automation, with such an automated sample preparation setup previously reported [43]. In summary, the analysis can be broken down into several steps, which are detailed below:

1. Change the concentration of the substrate;
2. Measure the change in light absorbance by the solution;
3. Determine the initial reaction rate;
4. Repeat (1)–(3) for different substrate concentrations;
5. Plot reaction rates versus substrate concentrations and determine *K_m_*.

### 3.2. Device Requirements and Architecture

The proposed miniaturised total analysis system should be automated, fully integrated (or not require external equipment), as accurate as commercially available equipment, compact and battery powered (to allow in situ operation), and finally, as low cost as possible. A key element of the device is the preparation of fluids for absorbance measurement. There are many options for the automated handling and preparation of fluids, such as fluid-handling robots, digital or pressure-driven fluidic systems, and microfluidic platforms using syringe pumps. A viable option, amenable to miniaturisation, is a pressure-driven fluidic system. *p*-Nitrophenol shows a peak in absorption at 407 nm for solutions with pH > 7 [44]. The sensor needs to detect a change in intensity at this wavelength with an accuracy of 5%. In addition, the sensor has to be in contact with the sample as this eliminates any light absorption or interference from the surrounding material.

**Figure 1:**
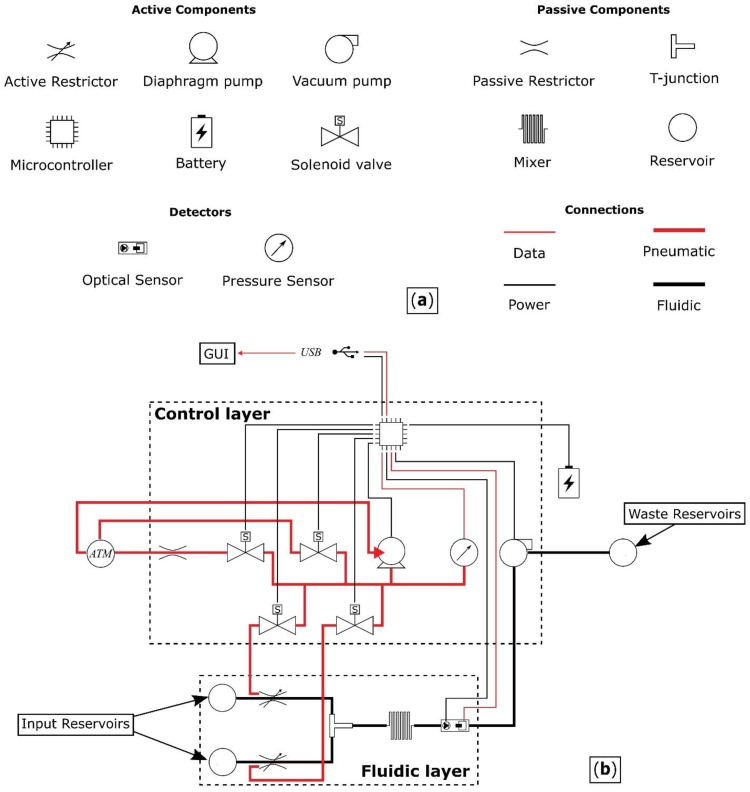
(**a**) Schematic representation of the passive and active components, detectors and connections between the components used in the design of the fluidic device. (**b**) Schematic of the proposed device architecture comprising a fluidic and a control layer.

The proposed device comprises a fluidic layer, where the solution is prepared and then optically measured, and a control layer, which contains the control circuitry of the system. Figure 1 shows a schematic of the device architecture, detailing all the components and their interconnections.

The components of the pressure-driven fluidic system are:

- Two reservoirs;
- Two active restrictors for controlling the flow rate from the reservoirs;
- A T-junction where the outputs following the restricted fluidic channels merge;
- A mixer;
- An analysis chamber, combined with a light source and an optical sensor for determining the initial reaction rate;
- Inlet/outlet ports;
- Interconnections.

Components can be active or passive. Fluids are driven from the reservoirs via the fluidic layer of Figure 1 using a pump to apply negative pressure, with waste being directed out of the system through an outlet port. The flow rate from each reservoir is controlled by restricting the diameter of the channels, thereby facilitating the use of different concentrations of *p*-nitrophenyl phosphate. The operating pressure range of the valves has to be greater than the pressure generated by the pump and the stiffness of the PDMS between the pressure chamber and the fluidic channel.

The size of the reservoirs is determined by the diameter and length of the fluidic components and the interconnecting channels, as well as the total number of experiments desired. The mixer ensures that the solutions are thoroughly mixed, but the enzymatic reaction must take place at the sensing component. Low pressures across the system can induce laminar flow, which is undesirable. For effective mixing, the mixer should promote turbulent behaviour. The diameter, cross-sectional topology and inner surface roughness of the interconnecting channels also determine the fluid dynamics.

The control layer provides a means for the automated operation of the fluidic device. It consists of an electronic circuit that powers and interacts with the device and a graphical user interface (GUI) that interacts with the user.

The electronic circuit uses a microcontroller unit (MCU) for acquiring data from the sensor, controlling the operational pressure of the two restrictive valves and moving the solutions through the fluidic device. Four solenoid valves are required for the operation of the two restrictive valves: two to direct the airflow to the pressure chambers, a third to vent the network to atmospheric pressure, and a fourth, with the addition of a restriction, to gradually reduce the pressure in the network for fine control. A pressure sensor is also required to control the restriction level of the fluidic channels.

The GUI enables the user to input the parameters of the experiment. These include the initial concentrations of the solutions present in the reservoirs, all the desired combinations of mixing ratios between the two solutions, and finally, the duration of the photometric measurement. The data are then collected and analysed to determine the enzyme kinetics. 

Once the system operation has been verified, full system automation in a single-unit system for use outside the laboratory simply requires the control scripts, desired experimental procedures, and parameters to be programmed directly to the MCU, thus eliminating the GUI and the computer. In such a case, user interaction would be much more constrained.

### 3.3. Design and Fabrication of the Fluidic Module

**Figure 2:**
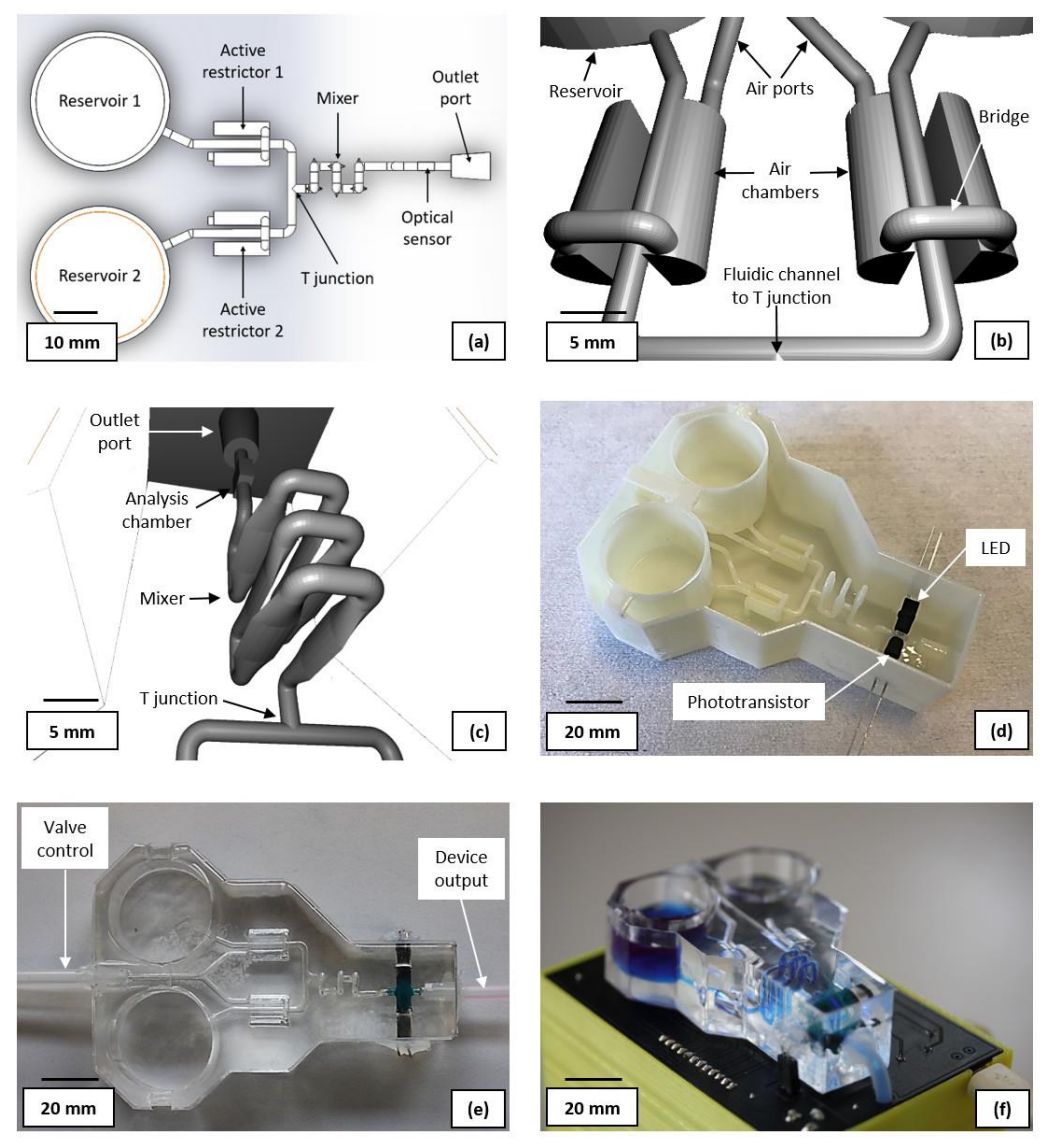
(**a**) Layout of the CAD for the sacrificial fluidic network; (**b**) 3D CAD of the proportional pneumatic valves (**c**) 3D CAD of the chaotic mixer; (**d**) 3D printed sacrificial mould, post acetone surface treatment with the LED and phototransistor attached; (**c**) PDMS fabricated fluidic device, with added fluidic interconnects to the control layer and waste reservoir; (**d**) the final device, integrating fluidics with the PCB and casing containing the battery, pumps, and valve system; (**e**) plan view of the fluidic device showing the pneumatic valve control tubes (left) and the liquid waste output tube (right), (**f**) photograph of the full system, filled with blue liquid to help visualise the liquid flow-path.

Figure 2a shows the layout of the computer-aided design (CAD) for the sacrificial fluidic mould (CAD available in the supplementary material). The reservoirs were sized as 20 × 25 mm (height × diameter) cylinders with a 1.5 mm (diameter) outlet channel at their base. This provides storage for adequate sample volumes for the anticipated number of experiments. The active restrictors were designed as proportional pneumatic valves consisting of a 1.5 mm diameter, 10 mm-long fluidic channel, flanked by two bridged semi-cylindrical air chambers of 5 mm diameter and 10 mm length. Both air chambers are connected via a single 1.5 mm diameter pressure inlet channel for control. The 3D CAD of the design can be seen in Figure 2b. By increasing the air pressure in the chamber, the PDMS moves towards the fluidic channel from both sides, reducing its cross-section. Further increasing the pressure will eventually fully obstruct the channel. A compact chaotic flow mixer was designed, based on six vertically oriented vesica piscis-shaped fluidic channels, each 3 mm high, 0.8 mm wide, and 10 mm long. The shift from a standard cylindrical channel to the described shape aims to assist with inducing a turbulent flow. To further promote a disturbed fluid flow and thus improve the mixing efficiency, the neighbouring modules have a 90° vertical rotational misalignment with each other. The design of the full mixer is presented in Figure 2c. The fluidic ports (outlet shown in Figure 2c) were designed as truncated cones (4 and 5 mm in diameter) with the larger diameter connecting to the external tubing.

The photometric sensor consists of a 3 × 3 × 1.5 mm analysis chamber (Figure 2c), a light emitting diode (LED), and a phototransistor placed on the opposite sides of the analysis chamber. A 400 nm LED (LED3-UV-400-300, BivarOpto) was selected as the best alternative to an optimal 407 nm light source. A visible light phototransistor (TEPT4400, Vishay, Malvern, PA, USA) with sufficient spectral sensitivity at 400 nm, was chosen as the photodetector (bill of materials available in the supplemental material). 

Figure 2d shows the ABS 3D-printed sacrificial mould, with the LED and phototransistor attached. Prior to component attachment, the mould was first sprayed with acetone and quickly dried with compressed air to smooth the surface. FDM printing yields parts with rough surfaces, which are unsuitable for achieving an efficient fluidic flow [39]. Both the LED and the phototransistor were protected by an opaque thermoplastic material and attached to the analysis chamber, with the acetone-soluble dicyanoacrylate. The photometric sensor was then encapsulated with 5 g of PDMS, which had been mixed with Silc Pig blue silicone pigment (Smooth-On) and cured in a convection oven at 60 °C for 30 min. This, compounded with the thermoplastic, ensures that no ambient light can reach the phototransistor and introduce measurement artefacts. Furthermore, it reduces light scattering interference from the LED that is not travelling through the analysis chamber and could otherwise reduce the sensitivity of the measurement or overwhelm the signal.

To fabricate the fluidic module, 50 g of Sylgard 184 (DowDuPont, Wilmington, DE, USA) with a ratio of 25:1 (base to catalyst) was prepared and then degassed. The recommended ratio by the manufacturer is 10:1. However, the stiffness of the PDMS was reduced [45,46] to allow the operation of the proportional valve with lower pressures, prolonging the lifetime of the device. The mould was filled with PDMS and placed in an oven at 60 °C for 2 h. Once cured, the device was immersed in an acetone bath for 72 h before flushing the liquified ABS. The device was then placed in a fume hood for 48 h to allow the acetone in the PDMS to evaporate. Finally, Sil-Poxy (Smooth-On, Inc., Macungie, PA, USA) was used to glue 4 mm silicone tubes to the ports of the device, one for the liquid output and one for controlling each pneumatic valve. Figure 2e shows the fabricated fluidic device.

### 3.4. Design and Fabrication of the Control Module

The fluidic module is controlled by an ATmega328P MCU (Microchip) integrated on a custom PCB, which was designed and fabricated in-house. The MCU drives the four solenoid valves, a piston air pump, as well as reading data from a digital gauge pressure sensor (SSCDANN015PG2A3, Honeywell, Charlotte, NC, USA). This in turn controls the pressure in the pneumatic valves, providing variable liquid channel restriction. Furthermore, it drives a peristaltic liquid pump, which is used for flowing and mixing liquids during an experiment or for a cleaning protocol. Finally, the MCU provides power to the LED and reads the output voltage from the phototransistor. An FTDI FT232R transceiver is used for data communication between the MCU and a computer via micro-USB. The selected options enable the use of Arduino libraries for Matlab. Power is provided via a 6 V NiMH 1300 mAh rechargeable battery, or alternatively, the PCB includes a DC power socket. The bill of materials and schematics of the PCB are available in the supplemental material. The fluidic module is positioned on top of the PCB with connections for the LED and the phototransistor, as well as through holes for the silicone tubing. Figure 2f shows the final miniaturised total analysis system, with the output fluidic tubing going through the PCB into the encased hardware compartment containing the pumps and solenoids.

A Matlab-based GUI was designed to provide the user with partial control of the device, as well as to deliver a high level of automation. The user inputs the required parameters for performing an enzymatic analysis. The parameters are detailed in Section 3.2. A complementary GUI has also been designed for the calibration of the pneumatic valves. The Matlab script associated with the experimental GUI uses the calibration data to adjust the pressure in the appropriate pneumatic valve and prepare the sample for measurement. It then retrieves the data from the photometric sensor, which can be exported for analysis.

## 4. Results and Discussion

### 4.1. Device Calibration

The device was calibrated by first characterising the photometric sensor and extracting a calibration curve, which was then used to characterise the pneumatic valves. Reference solutions of known original product concentration, and where the reaction had already occurred, were used.

**Figure 3:**
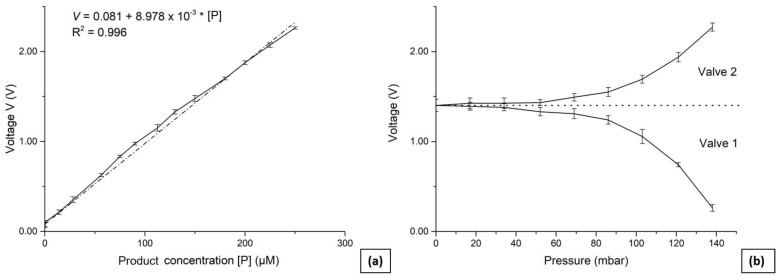
(**a**) Calibration curve of the photometric sensor showing a linear increase in output voltage with increased product concentration. (**b**) Calibration curve of the two pneumatic valves. The voltage readings were taken for different pressures applied in each active valve after the mixture of the two reservoirs was pumped in the analysis chamber.

The photometric sensor was calibrated by having a solution of known product concentration in the analysis chamber and then applying 5 V to the LED. Both the LED and the phototransistor are coupled with resistors that control the light intensity and output voltage linear range, respectively. The output voltage of the phototransistor was recorded every 100 ms for 30 s, and the mean value was calculated. This process was repeated by reducing the concentration via double dilution with a 500 mM Tris, 10 mM MgCl_2_ buffer (pH of 8.0) and until the output voltage matched this of a blank reading (buffer only). Product concentrations varied from 450 µM down to 0.9 µM. To maximise the sensor’s sensitivity and accurately measure the enzyme reaction rates across the widest possible range of concentrations, a number of resistors were tested during the development of the LED (68 Ω, 150 Ω, 220 Ω, 330 Ω, and 390 Ω) and the phototransistor (10 kΩ, 22 kΩ, 27 kΩ, and 33 kΩ). The sensor’s largest linear output voltage range of 2.152 V for concentrations between 250 µM and 0.9 µM, was observed when using a resistor of 150 Ω for the LED and 27 kΩ for the phototransistor. Using these resistor values, a calibration curve was extracted by taking five sets of measurements on 13 evenly distributed concentrations in addition to a blank reading. The sensor’s limit of detection (*LOD*) was then calculated using:

(3) $$LOD={\bar{x}}_{b}+3\times s_{b}$$

where ${\bar{x}}_{b}$ is the mean of the blank and $s_{b}$ is the standard deviation of the blank. For this work, ${\bar{x}}_{b}$ = 0.048 V and $s_{b}$ = 0.005 V, with a calculated *LOD* of 0.063 V, corresponding to 0.6 µM of *p*-nitrophenol. Figure 3a shows the calibration curve, whereas Table 1 summarises the sensor characteristics. The raw data for the sensor calibration are available in the supplementary material.

The calibration procedure for the pneumatic valves first involved filling reservoir 1 with 250 µM solution and reservoir 2 with a buffer. By operating the fluid pump for 2 s, the fluids were then mixed and transferred into the analysis chamber, prior to recording the output voltage of the calibrated photometric sensor. This was repeated with valve 1 (and similarly valve 2) pressurised at 17 mbar (5% of the pressure sensor range) and then incrementally in 17 mbar steps until the valve was fully shut at 155 mbar. The full procedure was repeated four times for both valves, and the mean values were extracted and plotted as calibration curves presented in Figure 3b. This enabled the correlation of channel flow rates and the pressure applied to the valves, and as can be seen, the valves behaved in a nonlinear manner. When the applied pressure is below 30 mbar there is no significant change in output voltage within the expected measurement error of the photometric sensor. The fluidic pump generates up to 600 mbar of suction pressure; therefore, 30 mbar of pressure at the valve is not sufficient to noticeably alter the flow.

### 4.2. Device Performance

To perform the enzyme kinetic analysis, reservoirs 1 and 2 were first filled with the substrate and enzyme solutions respectively. Six concentrations of substrate were used for the analysis ranging from 1390 µM to 40 µM, with the desired values entered via the GUI. The solution with the highest concentration of substrate can greatly exceed the range of the photometric sensor, as only initial reaction rates are necessary for the analysis. The Matlab script then retrieved the calibration data and pressurised the pneumatic valves according to the input parameters using the air pump. The liquid pump was then activated, and it successfully mixed and transferred the solutions into the analysis chamber. Measurements were taken for 300 s, and the output voltage from the photometric sensor was recorded every 100 ms. The data were then stored, and the script repeated the process for all substrate concentrations. All measurements were taken at room temperature. For subsequent measurements and in order to avoid the build-up of residuals through the channels and the detection chamber, the reservoirs were filled with isopropyl alcohol, which was then pumped through the device. This flushing protocol was typically repeated three times.

**Figure 4:**
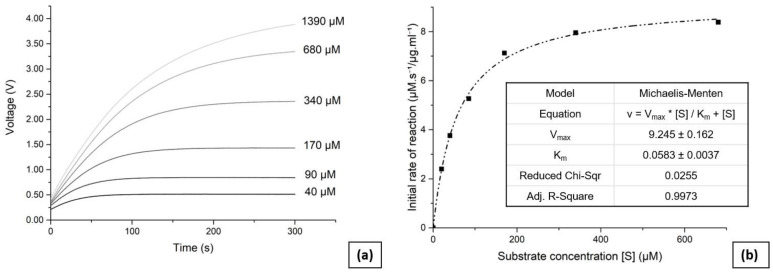
(**a**) Continuous measurement showing the output voltage of the photometric sensor over a period of 300 s. The graph displays the reaction rate for the six different concentrations of substrate used to perform the enzyme kinetics analysis. (**b**) Enzyme kinetics analysis using the Michaelis–Menten model, with *K_m_* = 58 µM ± 4 µM.

Figure 4a presents the obtained reaction progress curves as they were read and transmitted by the device to the GUI through the Matlab code, taken at varying initial substrate concentrations. The linear response of the photometric sensor enables the initial reaction rates to be determined without requiring a complex interpolation, thus simplifying the data analysis. The Matlab code calculated the initial reaction rate for each concentration of substrate, based on the initial slope (25 s) of the presented curves. Figure 4b presents the initial reaction rates plotted against substrate concentrations, with a curve fitted using a Michaelis–Menten fitting parameter. The enzyme kinetic value was determined to be *K_m_* = (58 ± 4) µM, which is consistent with the value of 60 µM reported previously [42,47].

### 4.3. Discussion

The device integrates pneumatics, fluidics, optics, and electronics. The fluidic module is 86 × 63 × 24 mm. The device is 109 × 74 × 88 mm, weighs 450 g, and includes a 105 g battery. The air pump and two solenoids are operated for up to 20 s while pressurising the pneumatic valves, whereas the liquid pump is operated for 2 s to flow the solutions through the mixer and into the analysis chamber. The device requires 35 mAh to perform the enzyme kinetics analysis. The power for the electronics, including the FTDI chip, MCU, pressure sensor and passive components, is supplied by the computer running the Matlab code. The rest of the components, pumps, and solenoids are powered by the battery, which allows for 58 h of continuous operation. Due to the small form factor, battery operation, and laptop connectivity, the device is suited for operation outside the lab environment.

For reference, the same enzymatic analysis was performed using a commercial Synergy HTX Multi-Mode Reader, where it was determined that *K_m_* = 80 µM ± 30 µM. The enzyme and substrate were mixed manually in a multi-well plate prior to loading the samples into the reader. This procedure could be problematic for time-critical enzyme kinetics, which may require upgrading the reader with non-standard dual injector modules that enable fast inject and read operations. The cost, size, and weight of a standard commercial absorbance microplate reader are considerably increased [48] in comparison to the presented fluidic system. However, it should be noted that a comparison between the devices would normally require an analysis of the full set of specifications. For example, commercial devices often offer temperature control, which is not the case for the presented device. This may be considered a limitation of the current system (although the system is compact and may be set up in a temperature-controlled box), as the rate of the enzymatic reaction could be influenced by possible temperature fluctuations during the time of analysis. However, with minor design modifications, a heater module with a temperature sensor could be integrated into the device and allow for temperature control. Nevertheless, the low cost, ease of manufacture, demonstrated accuracy, and ability for in situ operation, make the presented device a sufficient, and for some applications, desirable alternative to existing devices. Finally, it should be noted that a fully integrated and automated platform would also benefit from integrating artificial intelligence-assisted analysis into the system [49,50]. This is becoming increasingly important (e.g., biochemical analysis) and ultimately a desirable feature of such a system.

## 5. Conclusions

This paper presented the design, fabrication, and operation of a fully integrated miniaturised fluidic system. The device includes a pneumatic domain, an optic domain, a fluidic domain, and embedded electronics. With the addition of a custom control PCB and low-cost pumps, an enzyme kinetics analysis was demonstrated without requiring a dedicated lab, moving towards an autonomous lab-on-a-chip. The device was capable of producing accurate kinetic parameters for a well-characterised established biochemical assay. The system is low-cost, low-power, compact, and entirely portable for ad hoc use.

The fluidics of the device were fabricated using rapid prototyping techniques and are based on components which can be designed, modified, and characterised independently. This modular approach allows for easy adaptions in new designs and will allow to easily and rapidly create new fluidic devices that can perform a range of experiments.

## Figures and Tables

**Table 1:** The sensor characteristics extracted from the calibration curve in Figure 3a.

Range	Sensitivity	Limit of Detection
0.6–250 µM	8.978 mV/µM	0.6 µM

## Data Availability

The data presented in this study are available in the supplemental materials.

## Supplementary Materials

The following are available online at https://www.mdpi.com/article/10.3390/mi14030537/s1, Eagle files and BOM for the PCB, STL file of the mould of the fluidic device, raw data for the device’s calibration and raw data for the device comparative analysis with the plate reader.
