# Supplementary figures and images for: Design and Fabrication of a Fully-Integrated, Miniaturised Fluidic System for the Analysis of Enzyme Kinetics
Source: Micromachines (Basel). 2023 Feb 25;14(3):537. doi: 10.3390/mi14030537 (PMC10051508; doi:10.3390/mi14030537)

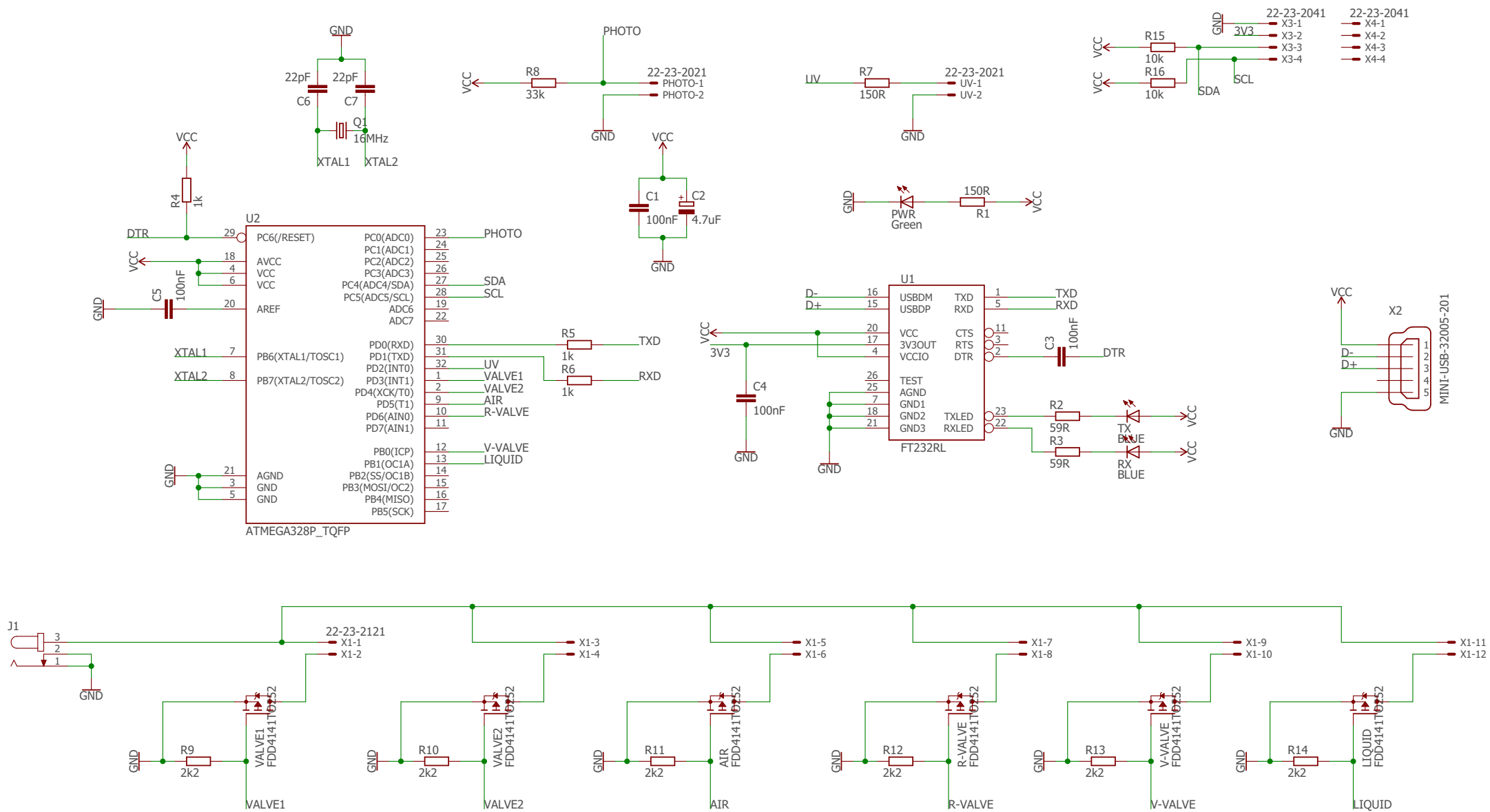

Supplement: Supplementary file 1 [file micromachines-14-00537-s001.zip › Supplemental Information/PCB/Eagle files/sch_1.pdf]
